# Factors Associated with Repeat Blood Donation at the Northern Zone Blood Transfusion Centre in Tanzania

**DOI:** 10.1155/2015/717653

**Published:** 2015-12-13

**Authors:** Wilhellmuss I. Mauka, Michael J. Mahande, Sia E. Msuya, Rune N. Philemon

**Affiliations:** ^1^Institute of Public Health, Department of Community Medicine, Kilimanjaro Christian Medical University College, P.O. Box 2240, Moshi, Tanzania; ^2^Northern Zone Blood Transfusion Centre, P.O. Box 823, Moshi, Tanzania; ^3^Institute of Public Health, Department of Epidemiology and Biostatistics, Kilimanjaro Christian Medical University College, Moshi, Tanzania; ^4^Department of Community Medicine, Kilimanjaro Christian Medical Centre, Moshi, Tanzania; ^5^Department of Pediatrics, Kilimanjaro Christian Centre, Moshi, Tanzania

## Abstract

*Background and Objective*. The aim of this study was to determine factors associated with repeat blood donation.* Methods*. This was a cross-sectional study carried out among blood donors aged 18–65 years in northern Tanzania. The questionnaire was administered among 454 participants through the phone.* Results*. Of the 454 participants, the proportion of repeat donation was 63.9%. In the backward logistic regression analysis, the significant predictors were living in Arusha which had lower odds of repeat donation compared to those living in Kilimanjaro. Knowledge of time interval between donations increased odds of repeating donations. High intention increased odds of repeat donation compared to low intention. Altruistic score had minor effect on increasing odds of repeating donation.* Conclusion*. Repeat blood donation is affected by proximity of donating site, awareness of the blood donation interval, intention to donate, and experience on previous donation. We recommend continuous education concerning blood donors and donation among health workers and society as a whole; this will create awareness on motivational factors for repeat donations.

## 1. Introduction

Blood donation is an act of a healthy person giving blood which will be used by another person in transfusion therapy and since it is a nonpharmaceutical product it has to come directly from a human being through donation [1, 2]. Globally, nearly 108 million units of blood are collected every year and half of those collections are from developed countries which are only one-fifth of total world population. High-income countries have at least 9 times more donations than low-income countries [3]. According to WHO's standards, country's minimum total blood donation collections should be 1% of total population in order to meet blood transfusion requirements [4].

According to Tanzania National Blood Transfusion Services (NBTS), by 2013, the country needed almost 450,000 units of blood annually. Between September 2013 and September 2014, a total of 133,077 units of blood were collected which is almost 30% of national requirement. However, more than 80% of blood donors donated only once and did not return for more donations (unpublished reports by Tanzania NBTS, 2013-2014). For the assurance of safe, adequate, and constant supply of the blood worldwide there is a need to retain blood donors who will become voluntary repeat regular donors [4].

Despite establishment of NBTS in 2004, there is a challenge in recruiting and retaining of the potential blood donors for repeating blood donations. Lack of repeat donors who donate blood regularly impedes blood collection which is to be planned systematically to meet the requirements of blood, by blood groups and components. Consequently, it incapacitates the blood transfusion service in maintaining a constant and reliable supply of safe blood when required in every clinical setting practicing transfusion [5].

There are several factors which have been associated with the return of blood donors for more donations. High intention and altruistic behavior have been pointed out to predict the repeat of blood donations [6, 7]. Knowledge concerning blood donation, convenient environment, and frequency of previous donation have been associated with repeat donations [8–10]. Sex and age have been associated with high turnover of repetition of donation whereby young males have higher chance of turning up for the repetition of blood donations than young females [9, 11].

To meet the demand for blood in the country, mobilization and sensitization activities have been commenced to recruit new and retain current blood donors. Because only a small proportion of eligible donors donate and an even smaller percentage return to give blood a second time or more; a better understanding of what motivates donors to give blood more than once is needed. This knowledge will help in planning, organizing, and implementation of donation activities in the country. Hence, the objective of this research was to determine different factors influencing repeat blood donation in our setting at Northern Zone Blood Transfusion Centre (NZBTC).

## 2. Methods

### 2.1. Study Design, Study Area, and Study Population

**Figure 1:**
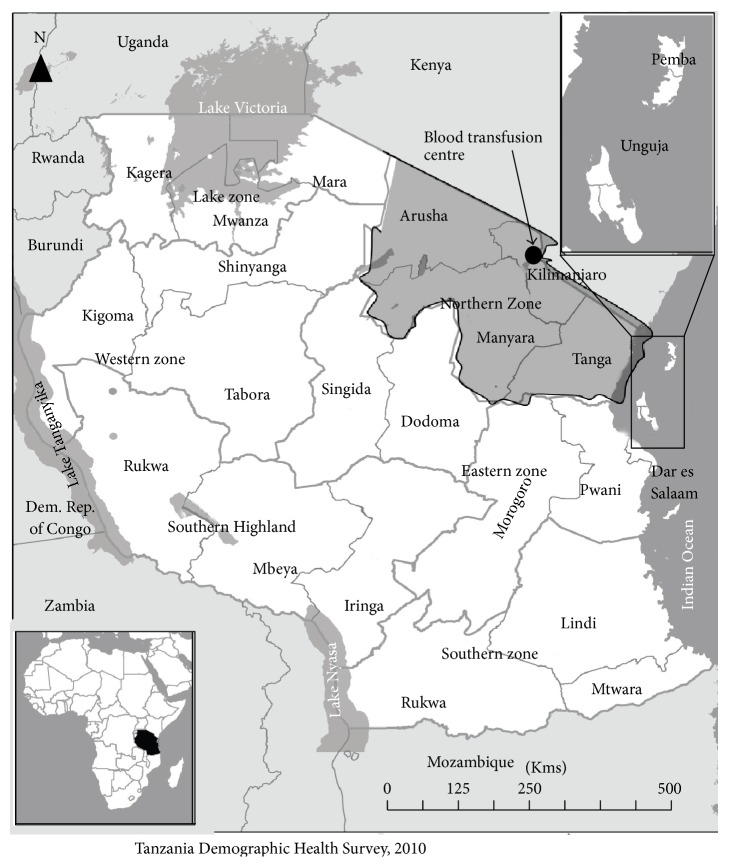
Location of the study area in Tanzania.

This was a cross-sectional study carried out between May 15 and June 15, 2015, at the Northern Zone Blood Transfusion Centre, Kilimanjaro, Tanzania. This centre is one of the six blood transfusion centers in Tanzania mainland, which serves four Northern regions in the country (Arusha, Kilimanjaro, Tanga, and Manyara) (Figure 1). The participants were blood donors registered between June 2012 and June 2014 in the donors' database, involving those who had donated once or more, aged 18 and above years, irrespective of gender and excluding those with no phone numbers.

### 2.2. Sample Size and Sampling

By using Kish Lisle formula for cross-sectional studies (Kish, 1967), the proportion of repeat donors was considered to be 50% in order to attain minimum sample size, with *Z*-score of 1.96 and standard error of 5%, plus 15% of nonresponse; a sample size of 442 participants was determined. But a total of 454 participants were recruited. Simple random sampling was employed whereby from donors' database (e-Delphyn Blood Bank software, version 5.7.0.0) registered between June 2012 and June 2014 and 34,115 donors were extracted. Only 12,969 (38%) who had telephone numbers documented were transferred into Microsoft Excel 2010, and RAND function was used to get random numbers which were reported in ascending order against the telephone numbers. Then participants were selected from top to bottom and contacted. A total of 460 participants were contacted and 454 agreed to participate making a response rate of 99%.

### 2.3. Data Collection

The participants were contacted through the phone and introduction of the study was done. For those who agreed to participate, date and time were set to conduct an interview at their convenience. The interview was conducted by four interviewers who were trained on questionnaire to have structured interview prior to the interview. By using questionnaire, the interview was done through the phone and the maximum time for interview was 20 minutes.

The participants were asked how many times they had donated blood in the past 5 years from the date of interview. The responses were ranging between 1 (once), 2 (twice), 3 (thrice), and 4 (more than 3 times). Those who reported more than 1 time were categorized as repeat donors [7]. The responses were not cross-checked with donors' database to confirm their responses.

Altruistic behavior score respondents were asked to score how often they had participated in the behavior from never (1) to very often (5); the responses were added to get a total score for each participant (minimum: 13; maximum: 65) [7]. Intention to donate in the next 12 months was reported from 1: “Very unlikely” to 5: “Very likely,” and those who were in between 1 and 3 were graded as low intention; those graded 4 and 5 had higher intention [7]. The same applied to those who reported their experience during last donation who had to score from 1 to 5 (Very bad–Very good). In analysis, those who scored 1 to 2 were graded as “bad experience” and those scored 3 to 5 were graded as “good experience.”

### 2.4. Data Analysis

Data was entered, cleaned, and analyzed with SPSS version 22 (IBM, NY, USA). Descriptive analysis was done whereby median for continuous data, frequency, and percentage were used to summarize the categorical data. Chi-square was used to test difference in categorical variables while Kruskal Wallis test was used for comparison of median scores of altruistic scores. The strength of association was determined by using crude and adjusted odds ratio (COR/AOR). Backward binary logistic regression model was employed to control the effect of confounding variables. *P* value less than 0.05 (2 tails) was considered statistically significant.

### 2.5. Ethical Consideration

Ethical clearance was sought and obtained from Kilimanjaro Christian Medical University College Research Ethical Committee. Permission was granted from National Blood Transfusion Service to access donors' database. Verbally informed consent was requested from each participant prior the interview. Participants identified details were not recorded on the questionnaire and collected information were used for this study purpose only.

## 3. Results

### 3.1. Sociodemographic Characteristics of Participants

A total of 454 blood donors participated in this study. The age of respondents was between 18 and 67 years. Median age was 29 (ranging between 24 and 39) years. Majority were males (72%). Most of respondents (54%) resided in Kilimanjaro region, followed by Arusha region (22%). At least half of respondents were single (50.9%). Primary education was mostly reported by respondents (38.8%) and the lowest proportion (0.7%) had no formal education. Profession-wise, the major group of respondents was public servants (21.1%) (Table 1).

Internal consistency of altruistic behavior scale (Cronbach's alpha, *α*) was 0.815. The minimum score was 18 and maximum was 65. The scores were unevenly distributed; hence, for more reliable measure of central tendency, median was employed, whereby the median was 47 (40–52).

**Figure 2:**
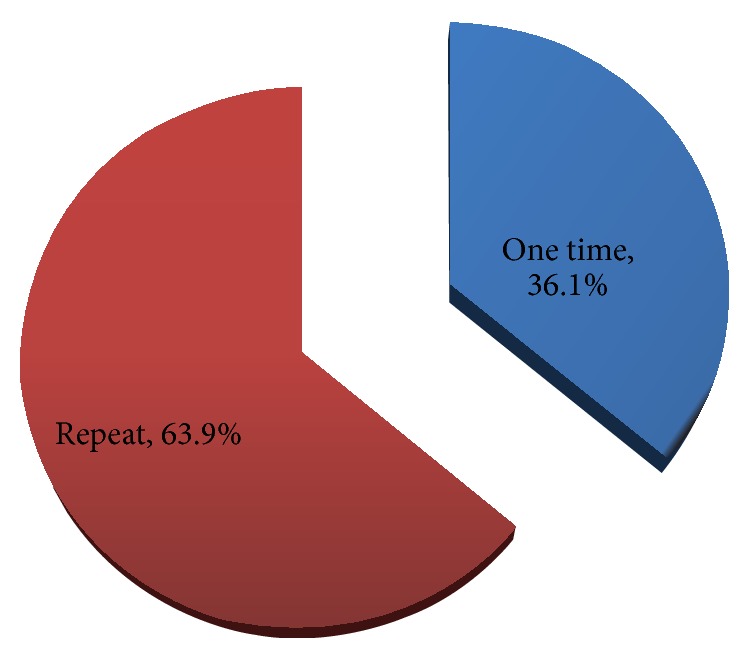
The proportion of donor's status.

Figure 2 shows the proportion of repeat donation which was 63.9%.

### 3.2. Factors Associated with Repeat Donation

Age and living place of the participants were significantly associated with donor status. Male had higher frequency (75.2%) of repeated donation compared to females (*P* = 0.047). Participants with secondary education (36.9%) had more repeated donations than others (*P* = 0.035). Majority (76.8%) had significant higher intention to donate compared to low intention (*P* = 0.000). Knowledge of donation interval (63.8 against 36.2%; *P* = 0.013) and good experience on last donation (61% against 39%; *P* < 0.001) had higher repeating donations prevalence compared to others (Table 2).

Majority of participants donated blood outside the blood centre, the reason being that the first donation was voluntary and for public use. Main concern or fear on their first donation was fainting while donating and majority were aged between 26 and 35 years. Most were public servants and single. Large proportion (96.9%) reported at least one usage of blood and had family member(s) who had donated blood.

**Figure 3:**
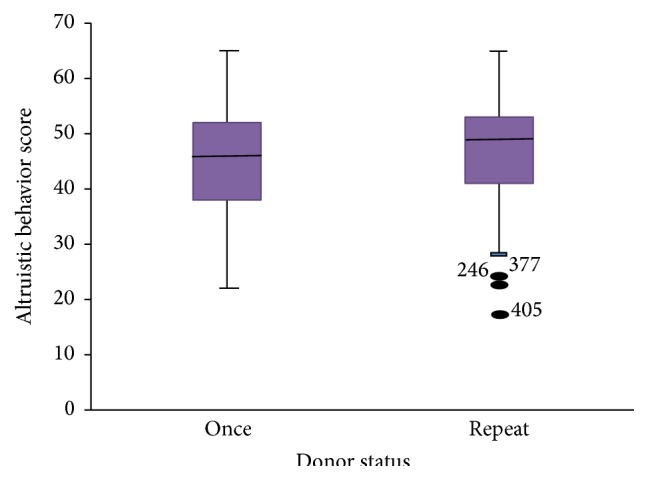
Altruistic behavior scores against donor status.

There was a significant difference in median scores of altruistic behavior among donor status, whereby the repeat donors tend to score higher than one time donors (*P* = 0.035, Mann-Whitney *U* test) (Figure 3).

In multivariate analysis, factors remained significant predictors of repeat donations. Those who were living in Arusha had lower odds of repeat donation by 0.466 times (AOR (95% CI); 0.47 (0.29–0.08)); *P* = 0.002; those living outside the Northern Zone had higher odds by 2.1-fold of repeat donations compared to those living in Kilimanjaro (AOR (95% CI) (1–4.34)); *P* = 0.049. Knowledge of time interval between donations increased odds of repeating donations by 55.4% than those who reported wrongly (AOR (95% CI): 1.52 (1.02–2.36)); *P* = 0.039. High intention increased odds of repeat donation by 2.1-fold (AOR (95% CI): 2.1 (1.34–3.24)); *P* = 0.001, compared to low intention. Good experience in previous donations increased odds of repeat donations (AOR (95% CI): 2.15 (1.42–3.25)); *P* < 0.001. Altruistic score had minor effect on increasing odds of repeating donations by 2.5% (AOR (95% CI): 1.03 (1–1.05)); *P* = 0.036 (Table 3).

## 4. Discussion

Proximity has a major role when it comes to motivating donors to return for more donations. The closeness of the donation site creates a convenient environment for the donors who are really intending to donate blood. This finding concurs with WHO strategy 16, on 100 % towards voluntary blood donation, in encouraging blood donation and repeat blood donation, whereby the long distance to the donation site may precipitate negative perception of inconvenience thus posing as barrier towards repeat donation [4]. Similar findings were reported by Schreiber et al., whereby majority of participants who could not routinely donate reported not having a convenient place to donate due to distance or transportation to the donation site [12].

In this study, majority of repeat blood donors reported high intention to donate in the next twelve months. Similarly, Godin and colleagues in their cohort study found that high intention was a strong predictor of returning for next donation [6]. Other literatures demonstrate that intention to donate is influenced by different factors such as sociodemographic characteristics [13, 14] and eligibility for donation [10]. In Zimbabwe, there has been an establishment of pledging system (Club 25) whereby the young people at the age of 16 are urged to pledge to donate blood almost 25 times in their life time [15]. This is one method of creating intention as in our social life pledging play a key role in making people commit to performing certain activity.

In the current study, awareness of time interval between donations was found to be significant information among repeat blood donors. Knowing the recommended time interval reminds the donors when to return for next donation. Similarly it has been reported by WHO that the donors who keep the pace or intervals of donation, as recommended by WHO [16], do develop habitual practice on the long run thus becoming potential voluntary repeat donors [6]. This leads to high frequency donation which has been demonstrated by other literatures, and that increases the likelihood of continuing to return for more donations [7, 8].

In our study, good experience in previous donation was significantly associated with repeat blood donation. It has been demonstrated by other studies that good handling of blood donors during the whole process of donation will increase the probability of returning of blood donors and building up the high intention to donate again [6, 8, 17]. Precounseling of blood donors on presumed adverse reactions may prepare the donors for unforeseen “bad experience.”

In the current study there was a weak significant association of altruistic behavior score and repeat donation. The same observations have been noted in other literatures [7, 8, 11, 18]. Although in our setting altruistic behavior score items [19] are reflected in day to day life and thus may not reflect the blood donation activity, encouraging message on building up altruistic motivation is advisable to create voluntarism atmosphere of helping one another in the society when it comes to blood.

*Strength and Weakness of the Study*. This is the first study conducted in Tanzania among blood donors on factors associated with repeat blood donation. Thus it can be used as baseline information for further studies.

Since participants were contacted and interviewed through the phone, study included only blood donors who had their mobile phone numbers recorded who represented 1/3 of the blood donors registered. Two-thirds of blood donors were excluded from the study as we did not have their mobile phone numbers recorded. Since their characteristics are not known, they might have different characteristics compared to those we studied. So the exclusion of those blood donors and their effect on our result cannot be quantified.

## 5. Conclusion

Blood donation is affected by proximity of donation site; thus living away from the donation centre decreases the chances of repeating donation. Establishment of several donation sites will encourage returning of blood donors. Knowledge of the blood donation interval has positive impact on repeating of donation; thus dissemination of such knowledge is crucial in retaining the potential donors. Higher intention of donating blood is the strong predictor of going back for more donations. Good previous experience motivates donors to return for more donations. Altruistic behaviors may have influence on repeat blood donation. We recommend continuous education concerning blood donors and donation among health workers and society as a whole. This will create awareness and motivational factors for repeat donations.

## Figures and Tables

**Table 1:** Sociodemographic characteristics of study participants.

Characteristics	Frequency (*n* = 454)	Percentage
Age (years)		
≤25	145	31.9
26–35	163	35.9
36–45	74	16.3
46–55	57	12.6
56 and above	15	3.3
Sex		
Male	327	72
Female	127	28
Area of residence (region)		
Kilimanjaro	248	54.6
Arusha	100	22
Tanga	44	9.7
Manyara	5	1.1
Outside Northern Zone	57	12.6
Marital status		
Single	231	50.9
Married	205	45.1
Others	18	4.0
Education Level		
No formal education	3	0.7
Primary	176	38.8
Secondary	155	34.1
College/university	120	26.4
Occupation		
Public servant	96	21.1
Private	81	17.8
Peasant	73	16.1
Business	79	17.4
Self-employed	44	9.7
Unemployed	11	2.4
Student	70	15.4

**Table 2:** Sociodemographic and donor characteristics of the participants by donor status (*N* = 454).

Characteristics	Repeated blood donation		
	*N*	*n* (%)	
Age group (years)			0.004
18–25	145	81 (27.9)	
26–35	163	106 (36.6)	
36–45	74	50 (17.2)	
46–55	75	38 (13.1)	
56–67	15	15 (5.2)	
Area of residence			0.002^*Ω*^
Kilimanjaro	248	161 (54.6)	
Arusha	100	50 (22)	
Tanga	44	29 (9.7)	
Manyara	5	5 (1.1)	
Outside NZ	57	45 (15.5)	
Sex			0.047
Male	327	218 (75.2)	
Female	127	72 (24.8)	
Level of education			0.035
No formal education	3	3 (1.0)	
Primary	176	99 (34.1)	
Secondary	155	107 (36.9)	
College/university	120	81 (27.9)	
Intention to donate			<0.001
Low	135	67 (23.2)	
High	318	222 (76.8)	
Knowledge on interval of blood donation			0.013
3-4 months	270	185 (63.8)	
Others	184	105 (36.2)	
Experience in last donation			<0.001
Bad	208	113 (39)	
Good	246	177 (61)	

^*Ω*^Fisher's Exact test otherwise *χ* ^2^ test.

**Table 3:** Factors associated with repeat donation among blood donors (*N* = 454).

Variables	Repeated donation			
	COR (95% CI)	*P* value	AOR (95% CI)	*P* value
Living area				
Kilimanjaro	1			
Arusha	0.54 (0.34–0.87)	0.01	0.47 (0.29–0.08)	0.002
Tanga	1.05 (0.53–2.05)	0.899		
Manyara	—	0.999		
Outside NZ	2.03 (1.02–4.03)	0.04	2.09 (1–4.34)	0.049
Intention to donate				
Low	1		1	
High	2.35 (1.55–3.55)	0.000	2.09 (1.34–3.24)	0.001
Donation interval				
Others	1		1	
3-4 months	1.64 (1.11–2.42)	0.01	1.56 (1.02–2.36)	0.039
Experience in last donation				
Bad	1		1	
Good	2.16 (1.46–3.18)	0.000	2.15 (1.42–3.25)	0.000
Altruistic score	1.03 (1.01–1.05)	0.01	1.03 (1–1.05)	0.036
